# Gliadin-Mediated Proliferation and Innate Immune Activation in Celiac Disease Are Due to Alterations in Vesicular Trafficking

**DOI:** 10.1371/journal.pone.0017039

**Published:** 2011-02-25

**Authors:** M. Vittoria Barone, Delia Zanzi, Mariantonia Maglio, Merlin Nanayakkara, Sara Santagata, Giuliana Lania, Erasmo Miele, Maria Teresa Silvia Ribecco, Francesco Maurano, Renata Auricchio, Carmen Gianfrani, Silvano Ferrini, Riccardo Troncone, Salvatore Auricchio

**Affiliations:** 1 Department of Pediatrics and European Laboratory for the Investigation of Food-Induced Diseases (ELFID), University of Naples Federico II, Naples, Italy; 2 Istituto di Scienze dell'Alimentazione, CNR Avellino, Avellino, Italy; 3 Laboratory of Immunological Therapy, Istituto Nazionale per la Ricerca sul Cancro, Genoa, Italy; Cornell University, United States of America

## Abstract

**Background and Objectives:**

Damage to intestinal mucosa in celiac disease (CD) is mediated both by inflammation due to adaptive and innate immune responses, with IL-15 as a major mediator of the innate immune response, and by proliferation of crypt enterocytes as an early alteration of CD mucosa causing crypts hyperplasia. We have previously shown that gliadin peptide P31-43 induces proliferation of cell lines and celiac enterocytes by delaying degradation of the active epidermal growth factor receptor (EGFR) due to delayed maturation of endocytic vesicles. IL-15 is increased in the intestine of patients affected by CD and has pleiotropic activity that ultimately results in immunoregulatory cross-talk between cells belonging to the innate and adaptive branches of the immune response. Aims of this study were to investigate the role of P31-43 in the induction of cellular proliferation and innate immune activation.

**Methods/Principal Findings:**

Cell proliferation was evaluated by bromodeoxyuridine (BrdU) incorporation both in CaCo-2 cells and in biopsies from active CD cases and controls. We used real-time PCR to evaluate IL-15 mRNA levels and FACS as well as ELISA and Western Blot (WB) analysis to measure protein levels and distribution in CaCo-2 cells.

Gliadin and P31-43 induce a proliferation of both CaCo-2 cells and CD crypt enterocytes that is dependent on both EGFR and IL-15 activity. In CaCo-2 cells, P31-43 increased IL-15 levels on the cell surface by altering intracellular trafficking. The increased IL-15 protein was bound to IL15 receptor (IL-15R) alpha, did not require new protein synthesis and functioned as a growth factor.

**Conclusion:**

In this study, we have shown that P31-43 induces both increase of the *trans*-presented IL-15/IL5R alpha complex on cell surfaces by altering the trafficking of the vesicular compartments as well as proliferation of crypt enterocytes with consequent remodelling of CD mucosa due to a cooperation of IL-15 and EGFR.

## Introduction

Celiac disease (CD) is characterised by derangement of both the adaptive and the innate immune responses to gliadin, that is a storage protein of wheat. Some gliadin peptides that are deamidated by tissue transglutaminase (e.g., A-gliadin P57-68) bind to HLA DQ2 and/or DQ8 molecules and induce an adaptive Th1 pro-inflammatory response [1]. There is also evidence that gliadin contains other peptides (i.e., P31-43) able to initiate a response involving innate immunity [2], [3].

Damage to the intestinal mucosa in CD is mediated both by inflammation due to the adaptive and innate immune responses (with IL-15 as a major mediator of the innate immune response) and by proliferation of crypt enterocytes as an early alteration of CD mucosa causing crypt hyperplasia [4]–[6]. The celiac intestine is characterised, in fact, by an inversion of the differentiation/proliferation program of the tissue with a reduction in the differentiated compartment, up to complete villi atrophy, and an increase of the proliferative compartment, with crypt hyperplasia [7], [8].

We previously investigated the early events of celiac disease and in particular the interaction between gliadin peptides and intestinal epithelial cells. We found that the so-called gliadin toxic peptide (P31-43) delays endocytic vesicle maturation and consequently reduces epidermal growth factor receptor (EGFR) degradation and prolongs EGFR activation, which in turn results in increased cell proliferation and actin modifications in celiac crypt enterocytes and in various cells lines [9]. P31-43 enters CaCo-2 cells and intestinal enterocytes, interacts with early endocytic vesicles [10], [11], reduces their motility and delays their maturation to late endosomes [10]. Taken together, this information points toward an effect of certain gliadin peptides, i.e., P31-43, on endocytic function and indicates epidermal growth factor (EGF) signalling as one of the major pathways in the celiac intestine.

The pro-inflammatory cytokine IL-15 is a major mediator of innate immune response in CD. In fact, IL-15 is higher in the lamina propria and the intestinal epithelium of untreated celiac patients as compared with treated patients and controls [3], [12], [13]. It induces differentiation of dendritic cells [14] and is also secreted by the intestinal epithelium [15]. Moreover, IL-15 affects the proliferation, localisation and function of intraepithelial lymphocytes (IELs) in the intestinal mucosa of CD patients [16]–[19].

Gliadin peptides 31-43 and 31-49 are not recognized by T cells and induce an innate immune response in the celiac mucosa [2]. P31-43-induced activation of various markers of the innate immune response is inhibited by neutralising anti-IL-15 antibodies [2]. IL-15 mediates P31-43-induced expression of the stress molecule MIC-A in enterocytes [3] and reproduces most of the epithelial modifications caused by gliadin in CD patients, including IEL migration [12]–[14]. IL-15 also exerts pleiotropic activity that ultimately results in immunoregulatory cross-talk between cells of the innate and adaptive branches of the immune response [20]. Moreover, IL-15 can induce proliferation in intestinal epithelial cells [21].

IL-15 expression is tightly regulated at both the transcriptional and post-transcriptional levels [22]–[24]. Although IL-15 transcripts are widely expressed, the IL-15 protein is seldom detected in the supernatants of cells that display mRNA for this interleukin [22], [24]. IL-15 has been found in the Golgi complex and in transferrin-carrying endocytic vesicles [25], [26]. Trafficking of the IL-15/IL-15R alpha complex in the endocytic pathway plays a central role in the regulation of IL-15 expression at the post-transcriptional level. IL-15 is chaperoned through the secretory pathway by complexing with IL-15 R alpha, as this complex forms in the Golgi and is transported to the membrane where it recycles and is *trans-*presented to neighbouring cells [27]–[31]. Interestingly, in the intestine, IL-15 is present on the surface of enterocytes, which suggests that cell-to-cell contact could play a role in IEL regulation [13].

The aims of this study were to determine if the proliferative activity of P31-43 on celiac enterocytes and cells is not only EGFR-dependent but also mediated by IL-15. We also investigated whether P31-43 increases IL-15 in an intestinal epithelial cell line (CaCo-2 cells) and the molecular and cellular bases of this phenomenon in relation to the derangement of the vesicular function induced by P31-43.

## Materials and Methods

### Cell culture, materials and transfections

CaCo-2 cells were grown for 5–6 days in Dulbecco's Modified Eagle's Medium (DMEM) (GIBCO, San Giuliano Milanese, Italy), 10% fetal calf serum (GIBCO), 100 units/ml penicillin-streptomycin (GIBCO), and 1 mM glutamine (GIBCO) with medium changed every two days.

Synthetic peptides were obtained from Inbios srl (Naples, Italy) and they were >95% pure as evaluated by matrix-assisted laser desorption/ionization time-of-flight mass spectrometry. Lipopolysaccharide (LPS)-free peptides were obtained by Ultrasart-D20 filtration (Sartorius AG, Gottingen, Germany)[9].

The levels of LPS in these peptides were below the detection threshold, i.e., <0.20 EU/mg as assessed with the QCL-1000 kit (Cambrex Corporation, NJ). The P31-43 sequence was LGQQQPFPPQQPY and the P57-68 sequence was QLQPFPQPQLPY. Dose/response experiments indicated that the best concentration of peptides for experiments involving bromodeoxyuridine (BrdU) incorporation and IL-15 expression on the cell surface (Fig. S1) was 100 µg/ml [9].

IL-15 PE-conjugated monoclonal antibody (clone: 34559; isotype: IgG1) was purchased from R&D Systems (Minneapolis, MN, USA). Rat isotype-matched PE-labelled control IgG1s were purchased from Pharmingen (San Diego, CA, USA). Recombinant human IL-15 (R&D Systems, Minneapolis, MN, USA) was used at a concentration of 10 ng/ml for FACS analysis and the blocking monoclonal anti-human IL-15 antibody (R&D Systems, Minneapolis, MN, USA) at 5 µg/ml in all experiments. We used the goat, anti-human IL-15R alpha (R&D Systems, Minneapolis, MN, USA), rabbit anti-human IL-15 (Santa Cruz Biotechnology, Santa Cruz, CA, USA) and mouse anti-alpha tubulin (Sigma-Aldrich, Milan, Italy) and rabbit anti-EGFR (Cell Signaling Celbio, Milan, Italy) antibodies for western blotting. BrdU was detected with a monoclonal antibody (GE Healthcare, Bickinghamshire, UK) and an anti-mouse-Alexa-488 conjugated secondary antibody (Molecular probes, San Giuliano Milanese, Italy). Nuclei were stained with Hoechst (Sigma-Aldrich, Milan, Italy). BrdU incorporation experiments to evaluate cell proliferation were carried out as described elsewhere.^9^ Blocking antibodies EGFR (528) (Santa Cruz Biotechnology, Santa Cruz, CA, USA) and IL-15 (R&D Systems, Minneapolis, MN, USA) were used at concentrations of 2 µg/ml and 5 µg/ml, respectively, for the BrdU assay.

Transfection of siIL-15R alpha was carried out following the manufacturer's instructions (QIAGEN) with HIPerFect Transfection Reagent. Briefly, CaCo-2 cells were incubated in standard growth conditions and 500 ng of IL-15R alpha siRNA were diluted in 100 μl of culture medium without serum to give a final siRNA concentration of 10 nM. Twenty microliters of HIPerFect Transfection reagent were added to the siRNA mix by vortexing. The transfection mix was added drop-wise onto the cells which were incubated for 72 h. Cells were than processed for WB or FACS analysis. Transfection of IL-15-EGFP was carried out as described before [25].

Over night (O/N) treatment is intended as 16 h treatment.

### IL-15 analysis

CaCo-2 cells were stimulated at 37°C with P31-43, P57-68, cycloheximide (Sigma-Aldrich, Milan, Italy) or with medium alone. After incubation, cells were removed from the dish by scraping on ice and plated in 96-well V-bottom plates (Costar Celbio, Milan, Italy). Cells were plated at a density of 1×10^5^ cells/well and were washed with PBS and analysed for surface or intracellular cytokine expression. Membrane cytokines were identified by labelling cells with PE-conjugated anti-IL-15 mAb for 30 min at 4°C. In the experiments to detect intracellular cytokines, 10 µg/ml brefeldin A (Sigma-Aldrich, Milan, Italy) was added to the incubation media for 3 h. Intracellular cytokines were identified as previously reported [32]. Finally, cells were read with a cytometer. Cycloheximide was used at a final concentration of 2 mM [33]. Dose-response curve was done for P31-43 stimulation to find optimal P31-43 concentration (Figure S1).

Some of the cells stimulated with P31-43 or medium alone were treated with acid buffer (2 mM glycine and 150 mM NaCl) for 10 min at 4°C and then labelled with PE-conjugated anti-IL-15 mAb [28]. Flow cytometry was carried out with a FACSCalibur system (BD Bioscience, San Diego, CA, USA) and the results were analysed with CellQuestPro software (BD Bioscience, San Diego, CA, USA).

### Immunoprecipitation

Lysates were prepared as described previously and protein concentration was measured with a Bio-Rad protein assay kit (Hercules, CA, USA) [9]. Equal amounts of cell lysates (2 mg protein/mL) were used for immunoprecipitation. IL-15R alpha was immunoprecipitated using the anti-IL-15R alpha goat polyclonal antibody (Santa Cruz Biotechnology, Santa Cruz, CA, USA). Proteins were immunoblotted with specific antibodies.

### Western blot

Briefly, CaCo-2 cells were starved overnight in DMEM containing 0.1% FBS and then stimulated with P31-43 for various intervals at 37°C. Cells were washed twice and resuspended in lysis buffer. Cell lysates were analysed by SDS-PAGE and transferred to nitrocellulose membranes (Whatman Gmbh, Dassel, Germany). The membranes were blocked with 5% non-fat dry milk and probed with anti-IL-15, anti-IL-15R alpha, anti-tubulin and anti-EGFR. Bands were visualised with the ECL system (GE Healthcare, Amersham, Buckinghamshire, UK). Band intensity was evaluated by integrating all the pixels of the band without the background, calculated as the average of the pixels surrounding the band [9].

### Organ culture studies

Biopsy fragments from the duodenum were obtained from five untreated patients with active CD and three controls (affected by gastroesophageal reflux) for organ culture studies. The protocol of the study was approved by the Ethical Committee of the University “Federico II”, Naples, Italy (ethical approval: C.E. n. 230/05). Informed written consent was obtained from all patients. The biopsy fragments were cultivated as reported elsewhere (for details, see Text S1) [9], [34].

### Statistical analyses

GraphPad Prism (GraphPad Software, San Diego, CA, USA) was used for statistical analysis and graphic representation. Statistical analysis of differences was performed with Student's *t*-test. A *p* value <0.05 was considered statistically significant.

## Results

### P31-43-induced proliferation depends on EGFR and IL-15 functions in CaCo-2 cells and in enterocytes of cultured biopsies from patients with active celiac disease

We previously demonstrated that P31-43 induces proliferation of fibroblasts (NIH 3T3 cell line) and of crypt enterocytes from cultured biopsies of CD patients with active disease but not from controls. This proliferation is mediated in an EGFR-dependent manner [9].

**Figure 1:**
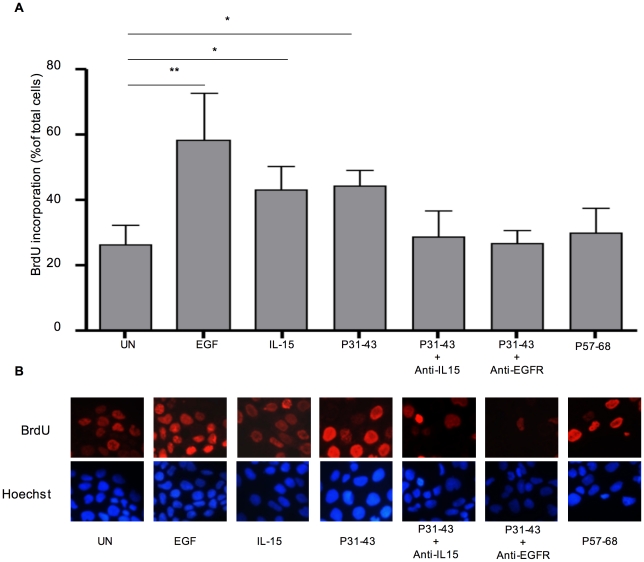
P31-43-induced EGFR- and IL-15-dependent proliferation in CaCo-2 cells. (A) Quantification of BrdU incorporation of CaCo-2 cells incubated overnight with medium alone, or treated as indicated. Columns represent the mean and bars represent the standard deviation of five independent experiments. More than 300 nuclei were counted for each experiment in several optical fields and the number of BrdU-positive cells was expressed as a proportion of the total nuclei. * = *p<*0.05 **p<0.01 (Student's *t*-test). (B) Immunofluorescence staining of BrdU incorporation of CaCo-2 cells treated as indicated. Hoechst stains of total nuclei. Single representative optical fields (63x objective).

We have now investigated whether P31-43 induces proliferation of an intestinal cell line such as CaCo-2 cells and whether this effect, as well as the P31-43 induced proliferation of celiac crypt enterocytes, is mediated not only by EGFR activation but also by IL-15 function. As shown in Fig. 1, not only EGF and IL-15 but also P31-43 induces proliferation of CaCo-2 cells, measured as the percentage of cells that incorporate BrdU. Treatment with P31-43 increased proliferation of CaCo-2 cells from 26.40%±5.7% in the untreated sample to 44.33%±4.5%. This proliferation is dependent on IL-15 and EGFR functions. In fact, both IL-15 and EGFR blocking antibodies reduced the percentage of proliferating cells to 28.57%±7.8% with IL-15-blocking antibodies and 26.67%±4% with EGFR-blocking antibodies. Similar results were obtained when CaCo-2 cells were treated with peptic-tryptic digest of gliadin (PTG, not shown). Peptide P57-78 had no effect on CaCo-2 cell BrdU incorporation.

**Figure 2:**
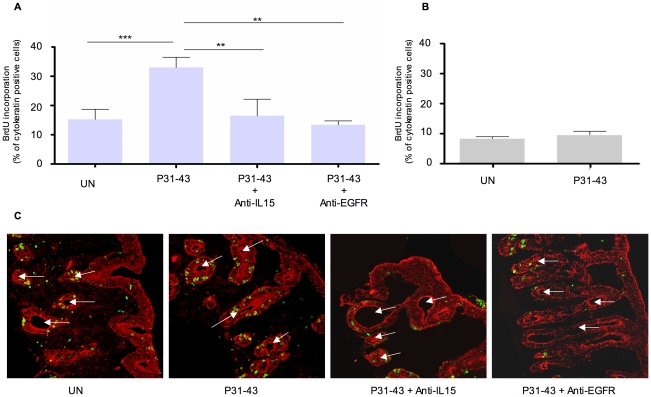
P31-43-induced proliferation of crypt enterocytes in celiac disease (CD) biopsies in the active phase of the disease depends on EGFR and IL-15 functions. (A) Quantification of BrdU incorporation of crypt enterocytes of intestinal biopsies from CD patients incubated with P31-43, with and without blocking antibodies anti-IL-15 and anti-EGFR. More than 300 cytokeratin-positive cells were counted in several fields in each sample and the number of BrdU-positive cells was expressed as a proportion of the total cytokeratin-positive cells. Mean and standard deviation of five independent experiments (Student's *t* test). ** = *p<*0.01; *** = *p<*0.001 (B) Quantification of BrdU incorporation of crypt enterocytes of intestinal biopsies from controls incubated with P31-43. More than 300 cytokeratin-positive cells were counted in several fields in each sample and the number of BrdU-positive cells was expressed as a proportion of the total cytokeratin-positive cells. Mean and standard deviation of three independent experiments (C) Immunofluorescence of crypts of duodenal biopsies from patients with active CD stained for cytokeratin to identify epithelial cells [red] and for BrdU [green]. Representative single optical field (40x objective). Lumen of the crypt is highlighted by white arrows. For methods, see supplementary material.

We next investigated whether, in biopsies from CD patients in the active state of the disease, P31-43-induced proliferation of enterocytes required IL-15 function. As expected, P31-43 induced a statistically significant increase in BrdU incorporation in crypt enterocytes from CD patients (Fig. 2A and C) [9]. Prevention of P31-43-induced proliferation was accomplished not only with the use of anti-EGFR blocking antibody (Fig. 2A and C), but also with IL-15-blocking antibody (Fig. 2A and C) [9]. In fact, after treatment with IL-15 blocking antibody, the percentage of BrdU-positive cells decreased from 33%±3.4% in the P31-43 treated sample to 16.5%±5.6%. Similar results were obtained when biopsies from active CD patients were treated with PTG (not shown). In control patients, neither P31-43 (Fig. 2 B) nor PTG (not shown) induced any proliferation [9].

Altogether, these data indicate that gliadin peptide-induced proliferation of CaCo-2 cells and of CD enterocytes is mediated by both IL-15 and EGFR activities.

### Effect of gliadin peptide P31-43 on transcriptional regulation of IL-15

**Figure 3:**
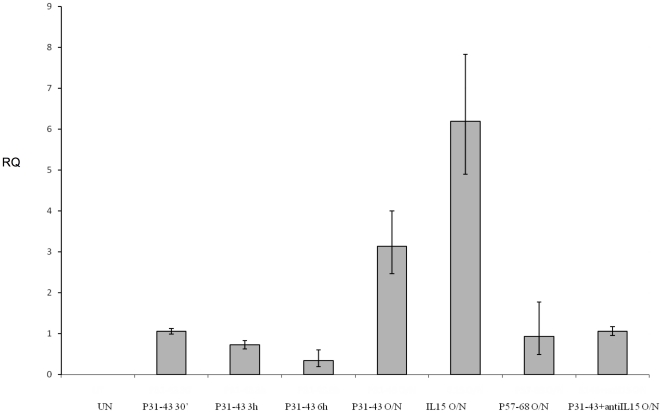
Overnight treatment with gliadin peptide P31-43, but not P57-68, increased levels of IL-15 mRNA in CaCo-2 cells. Quantitative PCR analysis shows an increase of IL-15 mRNA after O/N treatment of CaCo-2 cells with P31-43 but not after 30 min, 3 h and 6 h. This increase can be prevented by IL-15 blocking antibodies. RQ = relative quantity of IL-15 mRNA. Columns represent means, and bars are standard deviations of a representative experiment done in triplicate. Four separate experiments show similar results. UN = untreated. For methods, see supplementary material.

We treated CaCo-2 cells with P31-43 for 30 min, 3 h, 6 h or O/N to determine whether the peptide affected IL-15 mRNA levels. Quantitative PCR analysis showed an increase in IL-15 mRNA only after O/N treatment with P31-43, the control peptide P57-68 was not able to increase IL15 mRNA at the same levels (Fig. 3). Intriguingly, this increase in IL-15 mRNA is IL-15-dependent as it can be prevented by IL-15 blocking antibodies. This finding suggested that P31-43 acts on pre-existing IL-15 protein to further increase IL-15 mRNA accumulation in CaCo-2 cells. Indeed, exogenous IL-15 induced an even greater increase of IL-15 mRNA than did P31-43. (Methods are described in Text S2)

### P31-43 increased IL-15 protein expression on the surface of CaCo-2 cells, it did not do so in the cytoplasm or in the cell supernatant

**Figure 4:**
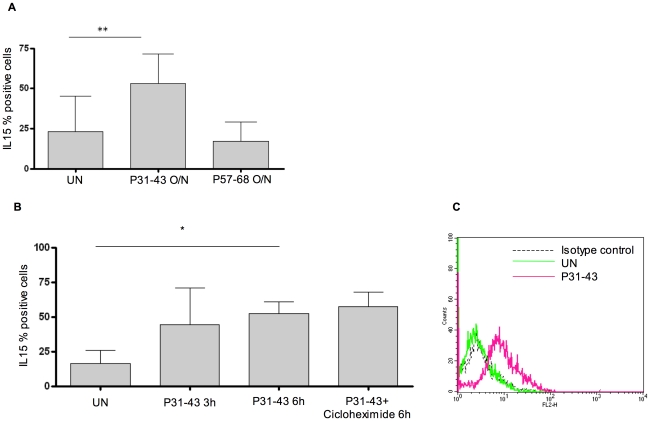
Gliadin peptide P31-43 increased IL-15/IL-15R alpha complex on the cell surface in CaCo-2 cells. (A) P31-43 increased IL-15 on the cell surface in CaCo-2 cells. FACS analysis of IL-15 on the cell surface after overnight (O/N), or 3 h or 6 h of treatment (B) with P31-43. Columns represent means and bars are the standard deviations of ten independent experiments for panel A and three independent experiments for panel B; * = *p<*0.05 (Student's *t*-test), ** = *p<*0.01 (Student's *t*-test) (C) Histogram of one representative experiment of CaCo-2 cells treated O/N with P31-43. Black dotted curve corresponds to negative control (isotype-matched Ab), the green open curve depicts specific IL-15 staining after medium treatment and pink open curve is specific IL-15 staining after O/N culture with P31-43.

To investigate whether P31-43 affects the expression of IL-15 protein, we evaluated (by FACS analysis) the intracellular and surface pools of IL-15 in CaCo-2 cells before and after exposure to P31-43. Overnight treatment with P31-43 did not affect the intracellular pool of IL-15 (Figure S2) and neither did shorter treatment times (not shown). We next evaluated whether P31-43 affects the extra-cellular release of IL-15 by CaCo-2 cells. After overnight incubation with P31-43, there was no statistically significant increase in IL-15 in the supernatant as measured by ELISA assay (Figure S3). However, the percentage of IL-15-positive cells on the surface increased from 22.92%±22.24% to 53.20%±18.26% after overnight treatment (Fig. 4A). This increase is specific for P31-43 because the control peptide P57-68 did not affect the percentage of cells expressing IL-15 on the surface (from 22.92%±22.24% to 17.09%±11.98%). IL-15 on the cell surface appeared to increase in expression after only 3 h of incubation with P31-43. This increase became statistically significant after 6 h of incubation, when it was comparable to that observed after overnight treatment (Fig. 4B). These findings indicate that either P31-43 increases the production of the IL-15 protein or mobilizes, from a pre-existing protein pool, IL-15 on the surface of the cells. We next analysed whether protein synthesis blockade induced by cycloheximide treatment was able to interfere with P31-43-induced increase of IL-15 on cell surfaces. Cycloheximide treatment failed to prevent the P31-43-mediated expression of IL-15 on the cell surface (57.42%+/−10.52% vs. 52.40+/−8.35, in the absence of cycloheximide) (Fig. 4B), suggesting that protein synthesis is not required for the P31-43 effect and that IL-15 is mobilized from an existing intracellular pool to the cell surface.

### Cell surface IL-15 is linked to IL-15R alpha

**Figure 5:**
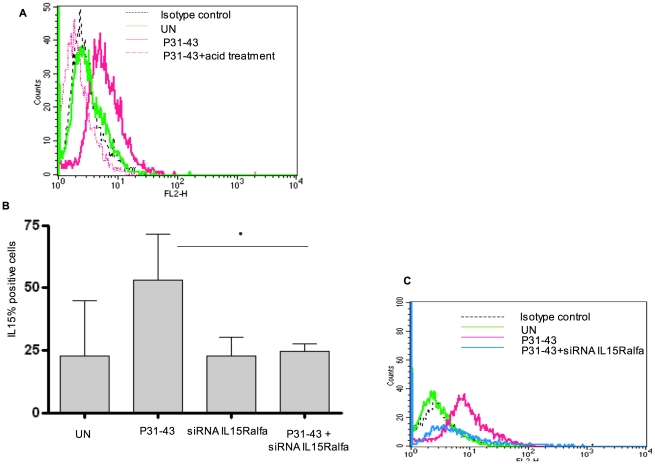
Acid treatment and siIl15 mRNA reduce the increase of IL-15 expression on CaCo-2 cell surfaces induced by P31-43. (A) FACS analysis of IL-15 on the cell surface after overnight (O/N) treatment with P31-43. Histogram of one representative experiment of CaCo-2 cells treated O/N with P31-43 and P31-43 plus acid treatment. Black dotted curve corresponds to negative control (isotype-matched Ab), the green open curve depicts specific mAb staining after medium treatment, pink open curve is specific mAb staining after O/N with P31-43 and the dotted pink open curve represents specific mAb staining after O/N with P31-43 plus acid treatment. Data are representative of one of five independent experiments. (B) siRNA IL-15R alpha reduces P31-43 mediated increase of IL-15 on CaCo-2 cell surfaces. FACS analysis of IL-15 on Caco-2 cell surfaces. Statistical analysis of ten independent experiments for UN (untreated) and P31-43 O/N treated cells and of four experiments for cells treated with siRNA IL-15R alpha. Columns represent means and bars are standard deviations. * = *p<*0.05 (Student's *t*-test). (C) Histogram of one representative experiment of CaCo-2 cells treated O/N with P31-43 and P31-43 plus SiRNA IL-15R alpha. The dotted black curve corresponds to the negative control (isotype-matched Ab), the green open curve depicts specific IL-15 staining after medium treatment, the pink open curve depicts specific IL-15 staining after treatment with P31-43 and the blue open curve represents specific IL-15 staining after O/N treatment with P31-43 in the presence of siRNA IL-15R alpha.

Duitman et al. demonstrated that membrane-associated IL-15 is directed to the cell surface in complex with IL-15R alpha, which serves as a chaperone for its ligand [30]. We therefore investigated whether cell surface IL-15, which is increased by P31-43, is also attached to the receptor in CaCo-2 cells (Fig. 5–7). PCR analysis confirmed the presence of IL-15R alpha mRNA in CaCo-2 cells (not shown).

**Figure 6 pone-0017039-g006:**
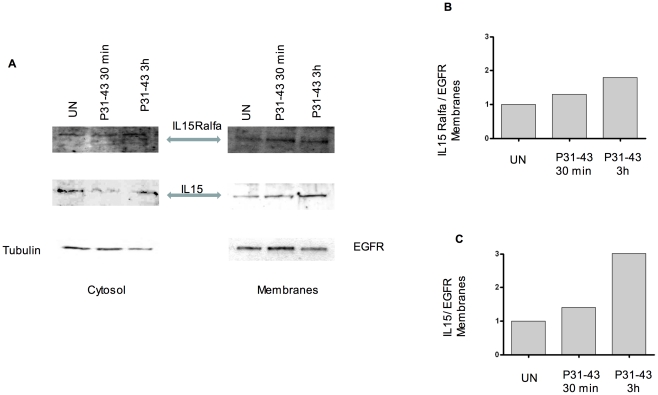
Both IL-15 and IL-15R-alpha expression increase in the isolated membrane fraction after stimulation with P31-43 for 30 min and 3 h. A) Western blot analysis of membrane proteins separated from total cell lysates shows an increase in membrane protein fractions of IL-15 and IL-15R alpha after P31-43 treatment. B and C) densitometric analysis of the Western blot experiment shown in a. EGFR was used to normalise membrane protein measurements. Increments (i) of IL-15 and IL-15R alpha were calculated as follows: iIL-15 =  (IL-15 treated [t]/IL-15 untreated [un])/(EGFR Treated [T]/EGFR Untreated [UN]). iIL-15R  = (IL-15R [t]/Il-15R [un]/(EGFR [T]/EGFR [UN]). The blots shown are representative of three similar independent experiments.

**Figure 7:**
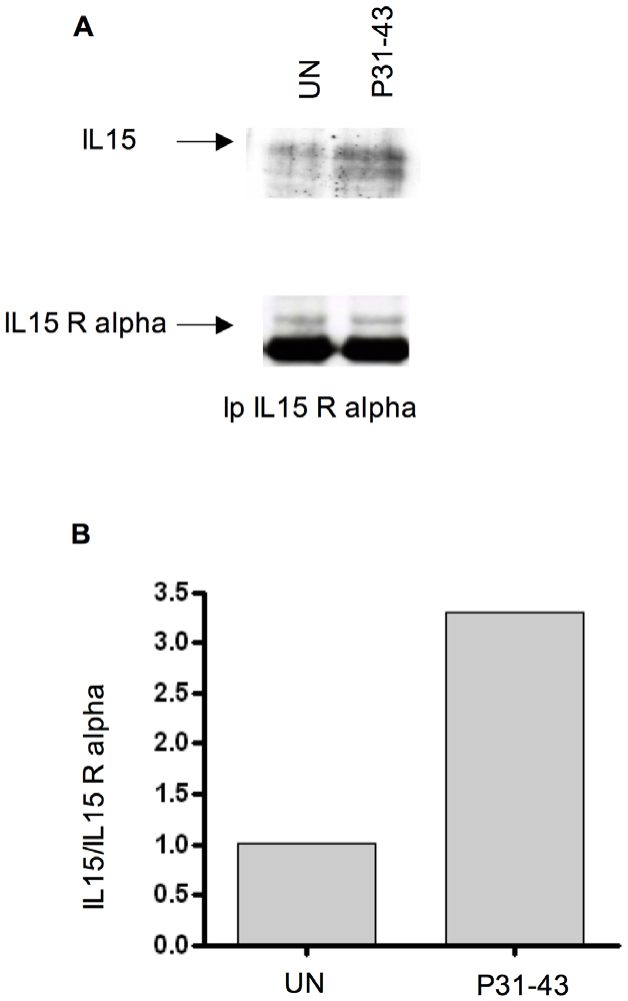
P31-43 treatment increases, on the cell membrane, IL-15/IL-15R alpha association. A) Demonstration of an IL-15/IL-15R alpha complex in an isolated membrane fraction. WB analysis of membrane proteins immunoprecipitated with IL-15R alpha antibodies. IL-15 and IL-15R alpha are visualized in the upper and lower gels with their respective antibodies. UN = untreated. B) Densitometric analysis of the Western blot experiment shown in (A). Fold increase of upper band of IL-15 (black arrow) was calculated respective to the IL-15R alpha band. The increment of IL-15 association (A) was calculated as follows: aIL-15 =  (IL-15 [t]/IL-15 [un])/(IL-15R alpha [T]/IL-15R alpha [UN]). The blot shown is representative of three independent experiments.

In addition, acid treatment known to release IL-15 from the ligand/receptor complex, reduced IL-15 on the surface of P31-43-treated CaCo-2 cells, suggesting that membrane-bound IL-15 is also linked to its receptor in this system (Fig. 5 A, B) [29]. To further confirm this hypothesis, we silenced IL-15R alpha by transfecting a specific siRNA, which reduced IL-15R alpha protein expression by almost 50% as shown in Figure S4. In Fig. 5 C,D the P31-43-induced increase of IL-15 on the cell surface is significantly inhibited (from 55%+/−18% to 24%+/−2.8%) in the presence of siIL-15R alpha. As expected, the increase of IL-15 on the cell membranes is mirrored by an increase in IL-15R alpha at the same site. In fact, both FACS analysis of the cells (not shown) and western blot analysis of proteins isolated from membrane fraction show an increase in IL-15 and IL-15R alpha after 30 min and after 3 h of treatment with P31-43 (Fig. 6). Finally, the existence of an IL-15/IL-15R alpha complex was demonstrated by analysis of proteins immunoprecipitated by anti-IL-15R alpha antibody from isolated cell membranes [29]. This analysis showed both immunoprecipitated IL-15 ligand and receptor by western blotting using specific antibodies (Fig. 7). Moreover, treatment with P31-43 increased the association of IL-15 and IL-15R alpha on the cell membrane by more than three-fold as compared to untreated cells when analysed by densitometry.

### P31-43-induced surface IL-15 is biologically active

**Figure 8:**
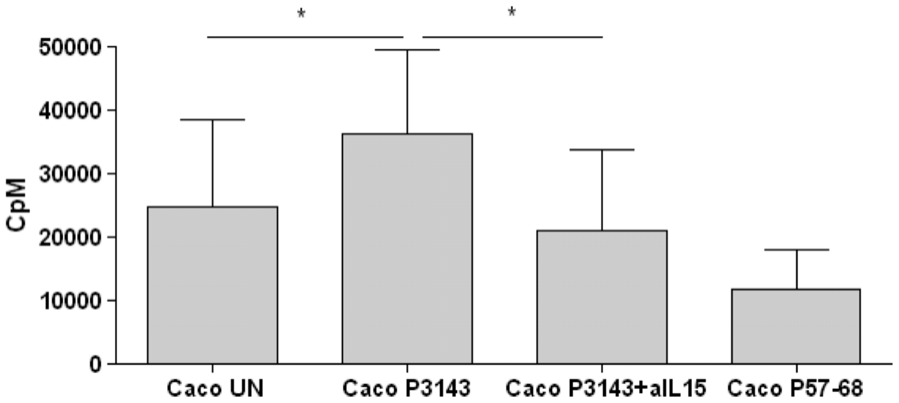
The complex IL-15/IL-15R alpha induced by P31-43 on the surface of CaCo-2 cells functions as a growth factor for CTLL2 cells. ^3^H-thymidine incorporation by CTLL2 cells induced to proliferate by CaCo-2 cells untreated or treated with P31-43 or P31-43 and anti-IL-15 or P57-68 was measured. CaCo-2 and CTLL2 cells were co-cultivated overnight. Data are expressed as ^3^H-TdR (CpM 1×10 ^6^ cells). Columns represent the mean, and bars represent the standard deviation of five independent experiments. *p<0.05 (Student's *t*-test). For methods, see supplementary material.

Most of the biological activity of IL-15 is believed to be mediated by the membrane-attached form of the protein [27], [28]. We therefore evaluated the functional activity of IL-15 on the CaCo-2 cell surface by co-culturing irradiated CaCo-2 cells, treated or not with P31-43, with CTLL2, a cell line responsive to the mitogenic effects of both IL-15 and IL-2 [35]. As shown in Fig. 8, the proliferation rate of CTLL2 cells, evaluated as ^3^H-thymidine incorporation, increased from 24,945 cpm±13,792 of the untreated sample, to 36,431 cpm±13,265 after P31-43 treatment of CaCo-2 cells. As expected, P57-68 treatment of CaCo-2 cells was not able to induce proliferation of CTLL2 (11,952+/−6,108). Furthermore, CTLL2 cells did not proliferate in response to direct treatment with P31-43 alone (not shown). The increase of ^3^H-thymidine incorporation was dependent on IL-15 because IL-15 blocking antibody treatment prevented CTLL2 proliferation induced by CaCo-2 cells treated with P31-43 (21,129+/−12,648). This finding indicates that IL-15 increased on the cell surface after P31-43 treatment can function as a growth factor. (Methods are described in Text S3)

### P31-43 alters trafficking of IL-15-containing recycling vesicles and increases recycling markers on CaCo-2 cell surfaces

**Figure 9:**
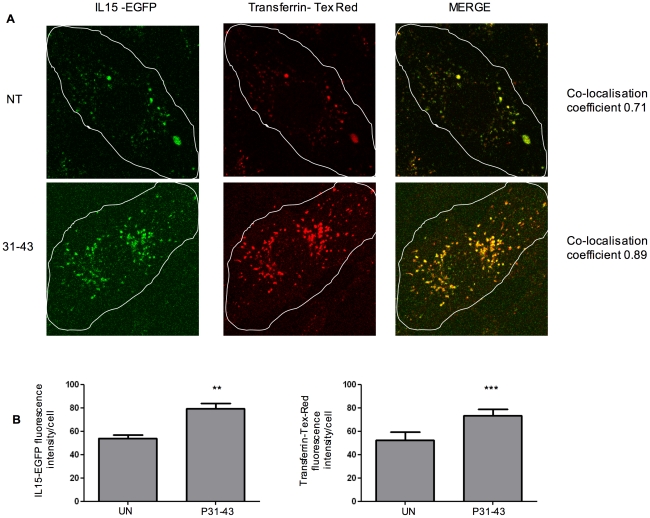
P31-43 alters trafficking of IL-15-containing recycling vesicles and increases recycling markers expressed on CaCo-2 cell surfaces. (A) IL-15-EGFP and Transferrin-Tex Red accumulate and co-localise after P31-43 treatment in a recycling vesicular compartment. IL-15-EGFP was transfected into CaCo-2 cells and observed by microscope after treatment with Transferrin-Tex Red and P31-43. White lines show the area of a single cell. (63x objective and 2x zoom). IL-15-EGFP (green) co-localises with Transferrin-Tex-Red (red) positive vesicles. Merge of the red and green panels is shown with yellow/orange colour indicating co-localisation. The co-localisation coefficient was calculated as reported under “Methods”. The results are representative of three independent experiments. For methods, see supplementary material. (B) Statistical analysis of fluorescence intensity/cell. For treated and untreated samples, three independent experiments were done, measuring fluorescence intensity of 10 cells in random fields in each experiment. ** = *p<*0.01, *** = *p<*0.001 (Student *t*-test). For methods, see supplementary material.

**Figure 10:**
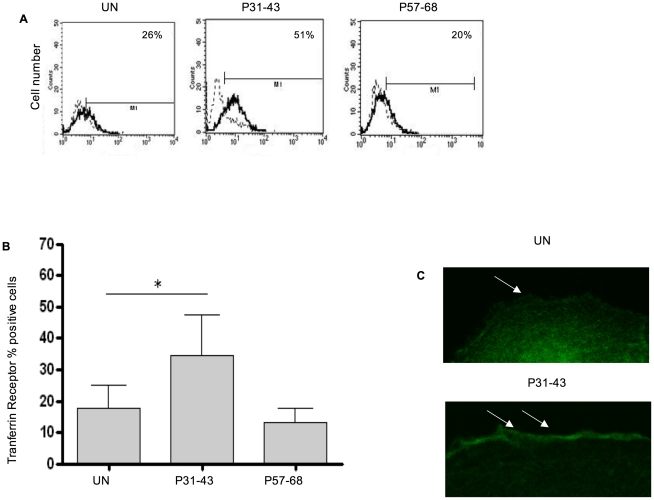
P31-43 increases expression of recycling marker transferrin receptor on the cell surface. A) FACS analysis of transferrin receptor, one experiment is shown. B) Statistical analysis of CaCo-2 cells percentage expressing the recycling marker, Transferrin Receptor, on the cell surface after P31-43 or P57-68 O/N treatment. Columns represent means and bars are the standard deviations of ten independent experiments. *p<0.05 (Student's *t* test). C) Confocal images of transferrin receptor expression on CaCo-2 cell surfaces. White arrows point to cell surface. 63x objective.

IL-15 has been found in the Golgi complex and in transferrin-carrying endocytic vesicles [25], [26]. We previously demonstrated that P31-43 alters the vesicular trafficking [9]. Therefore, we evaluated whether P31-43 affects the recycling pathway by carrying more IL-15 to the cell surface. IL15-EGFP localises to a recycling vesicular compartment when it is transfected in CaCo-2 cells [25]. After treatment with P31-43, IL-15EGFP-containing vesicles accumulated in the cytosol as shown in Fig. 9 A,B. The fluorescence intensity of the P31-43-treated cells exhibited a statistically significant increase from 54±2.9 to 79.3±4.7 after P31-43 treatment. To identify the IL-15EGFP-containing vesicular compartment, we treated CaCo-2 cells transfected with IL-15-EGFP with the recycling marker transferrin-Tex-Red for 90 min. [36]. As shown in Fig. 9A and B, treatment with P31-43 increased the number of transferring-containing vesicles, indicating that P31-43 can alter the trafficking of the recycling vesicles (fluorescence intensity/cell increased from 52.25±6.8 to 73.3±5.6 after P31-43 treatment). Treatment with P57-68 had no effect on the number of transferring-carrying vesicles. Furthermore, IL-15-EGFP co-localised with transferrin-Tex red in the same vesicular compartment before and after P31-43 treatment. To confirm P31-43 induced alterations of the recycling vesicular compartment, we investigated the levels of recycling marker transferrin receptor on the cell surface by FACS analysis before and after overnight treatment with P31-43 or P57-68. As shown in Fig. 10, the percentage of cells displaying the transferrin receptor on their surfaces significantly increased (from 18%±7% to 34.4%±13%) after P31-43 treatment while P57-68 treatment had no effect on the cell surface levels of transferrin receptor (from 18%±7% to 13%±4.7%). Therefore, P31-43 increased the expression of recycling vesicle markers on the cell surface, suggesting that the increase of IL-15 on the cell surface may relate to re-distribution of IL-15 from an intracellular vesicular compartment to the cell membrane. (Methods are described in Text S4.)

## Discussion

In this paper, we demonstrate that P31-43-induced cell proliferation both in crypt enterocytes and in CaCo-2 cells is not only dependent on EGFR but also on IL-15. P31-43 increased CaCo-2 cell surface expression of IL-15, the major mediator of innate immunity in CD, by altering the endocytic trafficking of the IL-15/IL-15R alpha complex. Thus, gliadin effects on proliferation and innate immunity activation are mediated by cooperation between growth factors (EGFR) and innate immunity mediators (IL-15) due to alterations in vesicular trafficking. It is now well accepted that endocytosis has many effects on signalling; in fact, signalling pathways and endocytic pathways are regulated in a reciprocal manner. It is also widely accepted that the “Endocytic Matrix” is a master organiser of signalling, governing the resolution of signals in space and time. Consequently, endocytosis affects several cell functions that range from proliferation to cell motility [37].

We first investigated the role of IL-15 in P31-43-induced cell proliferation. In previously published reports P31-43 has been found to be delayed in early endocytic vesicles both in crypt enterocytes of CD atrophic mucosa and in cell lines [9]–[11]. It has also been found that P31-43 can interfere with the correct localisation, on the vesicles surface, of the major coordinator of vesicle dynamics and maturation, namely the Hepatocyte growth factor Regulated tyrosine kinase Substrate (HRS) [10]. As a consequence maturation of the early endocytic compartment is delayed and the activation of EGFR and other receptors is prolonged, which results in several different biological events including cell proliferation [9]–[10].

In fact, the increase of proliferation of celiac crypt enterocytes induced by P31-43 was EGFR-dependent, as proliferation increase could be prevented by inhibitors of this pathway [9]. In this study, we show that IL-15, EGF and P31-43 or PTG can induce proliferation of an intestinal cell line such as CaCo-2. Moreover, we show that P31-43-induced proliferation is dependent on IL-15 and EGFR function. In fact, blockage of either the EGFR or IL-15 signalling pathways prevented P31-43-induced proliferation. These observations can be reproduced in intestinal biopsies from CD patients cultured for 24 hours. In this system, we show that PTG and peptide P31-43-induced crypt enterocyte proliferation is dependent not only on EGFR activation but also on IL-15 activity.

The present data and previously published reports [9] point to cooperation between a cytokine (IL-15) and a growth factor (EGF) to induce cellular proliferation. A complex between IL-15R alpha and EGFR is in fact present in CaCo-2 cells and is increased by P31-43 treatment (unpublished results). Such cooperation in signal transduction is not new. In fact, IL-15 and EGFR share the downstream effectors ERK and STAT [38]. IL-15 also interacts with the tyrosine kinase receptor AXL to prevent apoptosis in fibroblasts [39].

We next investigated whether gliadin-induced inflammation in CD is also affected by P31-43 alterations of the endocytic compartment. EGFR itself has a leading role in the regulation of the inflammation and can mediate innate immune responses in airway epithelium in respiratory diseases [40]. On the other hand, IL-15 is recognised as a major mediator of innate immunity in CD. In fact, it is not only increased in CD mucosa [3], [12], [13], but it is also necessary for the proliferation, localisation and function of intraepithelial lymphocytes (IELs) in the intestinal mucosa of CD patients [16]–[19]. Moreover, increased IL-15 activity mediates, to a large extent, the immune response induced by P31-43 in CD [2].

Therefore, we chose IL-15 activity as an indicator of the inflammation triggered by P31-43 and CaCo-2 cells as a model to study the capacity of P31-43 to increase IL-15 activity in an effort to understand the molecular mechanisms underlying this phenomenon.

IL-15 expression is tightly regulated at both transcriptional and post-transcriptional level [22]–[24].

Real-time PCR analysis showed that IL-15 mRNA increased in an IL-15-dependent manner only after prolonged incubation of CaCo-2 cells with P31-43, which suggests that the effects of gliadin on IL-15 mRNA could be secondary to other earlier effects. P31-43 increased IL-15 expression on the cell surface but not in the cytoplasm or at the level of protein secretion. The protein increase on the cell surface occurred earlier than the increase of IL-15 mRNA levels and independently from new protein biosynthesis, indicating that P31-43 first affected IL-15 protein distribution and then mRNA levels. Previous observations indicated that intracellular IL-15 localises to recycling vesicles that contain Transferrin Receptor and to the Golgi complex [20], [25]. Therefore, we investigated the effect of P31-43 on the early/endosomal vesicle recycling pathway. P31-43 treatment increased the number of vesicles carrying both IL-15-EGFP and Transferrin-Tex-Red.

Probably due to the accumulation of early endocytic vesicles induced by P31-43 [9]–[10] and to the delay of the maturation of this compartment to lysosomes [10], [11]. This could explain the increase of fluorescence for IL15 in absence of increased IL15 protein synthesis. Moreover, FACS analysis showed that P31-43 increases a typical marker of recycling vesicles, such as the Trasferrin Receptor, on the cell surface. These data are consistent with the hypothesis that P31-43-induced alteration of the endocytic pathway may be responsible for the increase of IL-15 expression on the cell surface.

It has recently been demonstrated that IL-15 is transported to the cell surface as a complex with its receptor, IL-15R alpha, which functions as a chaperone for the ligand, through the Golgi apparatus. This complex represents the *trans* form of IL-15 and enables the trafficking of this cytokine through the secretory and recycling pathways [30]. Here, we have shown that both IL-15 and IL-15R alpha increase in isolated membrane fractions of CaCo-2 cells after stimulation with P31-43. In addition, we showed, by immunoprecipitation, the presence of an IL-15/IL-15R alpha complex in the membrane fraction of CaCo-2 cells increased by P31-43 treatment. Finally, acid treatment and siRNA anti-IL-15R alpha reduce the amount of IL-15 present on the cell surface. Taken together, these data demonstrate that cell surface IL-15 is linked to the receptor.

The IL-15/IL-15R alpha complex present on the surface of CaCo-2 cells after P31-43 treatment is a functional growth factor for IL-15-sensitive CTLL2 cells. The membrane-bound, *trans*-presented IL-15 performs a number of IL-15 primary functions [27], [28]. In non-immune cells, *trans*-presented IL-15 protects fibroblasts and epithelial cells from apoptosis and induces their proliferation [21]. It is also capable of inducing angiogenesis, of mediating anabolic effects in muscle cells, and of stimulating the lipolysis of adipocytes and the survival of neuronal cells [27]. IL-15 induces these effects by activating intracellular pathways directly by cell-to-cell contact [28].

In conclusion, we have shown that P31-43 induces at least two main effects by altering the trafficking of cell vesicular compartments. This leads to overexpression of the *trans*-presented IL-15/IL5R alpha complex, an activator of innate immunity, and, due to cooperation of IL-15 and EGFR, the proliferation of crypt enterocytes with consequent remodelling of the CD mucosa.

These observations are relevant to our understanding of the early events occurring in the celiac mucosa exposed to gliadin because the increase of IL-15 and IL-15R alpha is a major event in the initial phases of CD [3], [12], [13], [41]. Our observation that in the celiac intestine IL-15 plays a major role in the gliadin-induced proliferation of epithelial cells, one of the hallmarks of CD, reinforces the importance of our results obtained in CaCo-2 cells and CD biopsies, which may increase understanding of the pathogenesis of CD. Why the celiac mucosa seems to be particularly sensitive to the effects of some gliadin peptides, such as peptide P31-43, remains to be elucidated. Preliminary data suggest that in CD cells, the endocytic compartment is morphologically and functionally altered. We hypothesize that in CD mucosa, an alteration of the vesicular compartment renders the tissue more sensitive to the effects of gliadin.

## Supporting Information

Figure S1 **Dose-response effect of P31-43 treatment on IL-15 expression on CaCo-2 cell surfaces FACS analysis of IL-15 on Caco-2 cells surfaces after O/N treatment with varying concentrations of P31-43 peptide.** UN = untreated. Columns indicate percentage of positive cells (mean and standard deviation of three independent experiments). *p<0.05 (Student's t-test). Optimised concentration of P31-43 for IL-15 expression on cell surface was 100 µg/ml.
(TIF)

Figure S2 **Overnight treatment with gliadin peptide P31-43 does not increase intracellular IL-15 expression.** FACS analysis of IL-15 in the cytoplasm of CaCo-2 cells. Columns indicate percentage of positive cells (mean and standard deviation of four independent experiments).
(TIF)

Figure S3 **Overnight treatment with gliadin peptide P31-43 does not increase secreted IL-15.** ELISA assay of IL-15 in medium of cultured CaCo-2 cells. Columns indicate pg/ml (mean and standard deviation of three independent experiments).
(TIF)

Figure S4 **siRNA IL-15R alpha reduces IL-15R alpha protein expression.** (A) CaCo-2 cells were transfected with IL-15R alpha siRNA, lysed and immunoblotted for IL-15R alpha expression. β-Tubulin was used as an internal control. (B) Densitometric analysis of IL-15R alpha expression compared to alpha-tubulin expression. The decrease (d) of IL-15R alpha was calculated as follows: dIL-15R  =  (IL-15R [t]/IL-15R [un])/(Tubulin [T]/Tubulin [UT]). Shown is one representative experiment out of three independent experiments.
(TIF)

Text S1 **Organ Culture Study.**
(RTF)

Text S2 **RNA Extraction and Real-Time PCR.**
(RTF)

Text S3 **CTLL2 Proliferation Assays.**
(RTF)

Text S4 **Transferrin and Transferrin Receptor Analysis.**
(RTF)

## References

[1] Sollid LM (2000). Molecular basis of celiac disease.. Annu Rev Immunol.

[2] Maiuri L, Ciacci C, Ricciardelli I, Vacca L, Raia V (2003). Association between innate response to gliadin and activation of pathogenic T cells in coeliac disease.. Lancet.

[3] Hue S, Mention JJ, Monteiro RC, Zhang S, Cellier C (2004). A direct role for NKG2D/MICA interaction in villous atrophy during celiac disease.. Immunity.

[4] Marsh MN (1993). Clinical and pathological spectrum of coeliac disease.. Gut.

[5] Marsh MN, Crowe PT (1995). Morphology of the mucosal lesion in gluten sensitivity.. Baillieres Clin Gastroenterol.

[6] Marsh MN, Loft DE, Garner VG (1992). Time dose responses of coeliac mucosae to graded oral challenges with. Frazer's Fraction III (FF3) of gliadin. Europ3′ Gastroenterol.. Hepatol.

[7] Diosdado B, Wapenaar MC, Franke L, van Oort E, Mulder CJ (2004). A microarray screen for novel candidate genes in coeliac disease pathogenesis.. Gut.

[8] Juuti-Uusitalo K, Maki M, Kainulainen H, Isola J, Kaukinen K (2007). Gluten affects epithelial differentiation-associated genes in small intestinal mucosa of coeliac patients.. Clin Exp Immunol.

[9] Barone MV, Gimigliano A, Castoria G, Maurano F, Paparo F (2007). Growth factor-like activity of gliadin, an alimentary protein: implications for coeliac disease.. Gut.

[10] Barone MV, Nanayakkara M, Paolella G, Maglio M, Vitale V (2010). Gliadin peptide P31-43 localises to endocytic vesicles and interferes with their maturation.. PloS One.

[11] Zimmer KP, Fischer I, Mothes T, Weissen-Plenz G, Schmitz M (2010). Endocytotic Segregation of Gliadin Peptide 31-49 in Enterocytes.. Gut.

[12] Maiuri L, Ciacci C, Auricchio S, Ricciardelli I, Vacca L (2000). Interleukin 15 mediates epithelial changes in celiac disease.. Gastroenterology.

[13] Mention JJ, Ben Ahmed M, Begue B, Barbe U, Verkarre V (2003). Interleukin 15: a key to disrupted intraepithelial lymphocyte homeostasis and lymphomagenesis in celiac disease.. Gastroenterology.

[14] Harris KM, Fasano A, Mann DL (2010). Monocytes differentiated with IL-15 support Th17 and Th1 responses to wheat gliadin: implications for celiac disease.. Clin Immunol.

[15] Di Sabatino A, Ciccocioppo R, Cupelli F, Cinque B, Millimaggi D (2006). Epithelium derived interleukin 15 regulates intraepithelial lymphocyte Th1 cytokine production, cytotoxicity, and survival in coeliac disease.. Gut.

[16] Maiuri L, Ciacci C, Vacca L, Vacca L, Raia V (2001). IL-15 drives the specific migration of CD94+ and TCR-gammadelta+ intraepithelial lymphocytes in organ cultures of treated celiac patients.. Am J Gastroenterol.

[17] Ebert EC (2005). IL-15 converts human intestinal intraepithelial lymphocytes to CD94 producers of IFN-gamma and IL-10, the latter promoting Fas ligand-mediated cytotoxicity.. Immunology.

[18] Meresse B, Chen Z, Ciszewski C, Tretiakova M, Bhagat G (2004). Coordinated induction by IL15 of a TCR-independent NKG2D signaling pathway converts CTL into lymphokine-activated killer cells in celiac disease.. Immunity.

[19] Kinoshita N, Hiroi T, Ohta N (2002). Autocrine IL-15 mediates intestinal epithelial cell death via the activation of neighboring intraepithelial NK cells.. J Immunol.

[20] Fehniger TA, Caligiuri MA (2001). Interleukin 15: biology and relevance to human disease.. Blood.

[21] Reinecker HC, MacDermott RP, Mirau S, Dignass A, Podolsky DK (1996). Intestinal epithelial cells both express and respond to interleukin 15.. Gastroenterology.

[22] Bamford RN, Battiata AP, Waldmann TA (1996). IL-15: the role of translational regulation in their expression.. J Leukoc Biol.

[23] Meazza R, Verdiani S, Biassoni R, Coppolecchia M, Gaggero A (1996). Identification of a novel interleukin-15 (IL-15) transcript isoform generated by alternative splicing in human small cell lung cancer cell lines.. Oncogene.

[24] Meazza R, Gaggero A, Neglia F, Basso S, Sforzini S (1997). Expression of two interleukin-15 mRNA isoforms in human tumors does not correlate with secretion: role of different signal peptides.. Eur J Immunol.

[25] Gaggero A, Azzarone B, Andrei C, Mishal Z, Meazza R (1999). Differential intracellular trafficking, secretion and endosomal localization of two IL-15 isoforms.. Eur J Immunol.

[26] Barzegar C, Meazza R, Pereno R, Pottin-Clemenceau C, Scudeletti M (1998). IL-15 is produced by a subset of human melanomas, and is involved in the regulation of markers of melanoma progression through juxtacrine loops.. Oncogene.

[27] Budagian V, Bulanova E, Paus R, Bulfone-Paus S (2006). IL-15/IL-15 receptor biology: a guided tour through an expanding universe.. Cytokine Growth Factor Rev.

[28] Bulfone-Paus S, Bulanova E, Budagian V, Paus R (2006). The interleukin-15/interleukin-15 receptor system as a model for juxtacrine and reverse signaling.. Bioessays.

[29] Dubois S, Mariner J, Waldmann TA (2002). IL-15Ralpha recycles and presents IL-15 In trans to neighboring cells.. Immunity.

[30] Duitman EH, Orinska Z, Bulanova E Tagaya Y (2008). How a cytokine is chaperoned through the secretory pathway by complexing with its own receptor: lessons from interleukin-15 (IL-15)/IL-15 receptor alpha.. Mol Cell Biol.

[31] Huntington ND, Legrand N, Alves NL, Jaron B, Weijer K (2009). IL-15 trans-presentation promotes human NK cell development and differentiation in vivo.. J Exp Med.

[32] Prussin C, Metcalfe DD (1995). Detection of intracytoplasmic cytokine using flow cytometry and directly conjugated anti-cytokine antibodies.. J Immunol Methods.

[33] Abou Elela S, Nazar RN (1997). Role of the 5.8S rRNA in ribosome translocation.. Nucleic Acids Res.

[34] Barone MV, Caputo I, Ribecco MT, Maglio M, Marzari R (2007). Humoral immune response to tissue transglutaminase is related to epithelial cell proliferation in celiac disease.. Gastroenterology.

[35] Paxton RJ (2001). Measurement of interleukin 15.. Curr Protoc Immunol.

[36] Maxfield FR, McGraw TE (2010). Endocytic recycling.. Nat Rev Mol Cell Biol.

[37] Scita G, Di Fiore PP The endocytic matrix.. Nature.

[38] Yano S, Komine M, Fujimoto M, Okochi H, Tamaki K (2003). Interleukin 15 induces the signals of epidermal proliferation through ERK and PI 3-kinase in a human epidermal keratinocyte cell line, HaCaT.. Biochem Biophys Res Commun.

[39] Budagian V, Bulanova E, Orinska Z, Thon L, Mamat U (2005). A promiscuous liaison between IL-15 receptor and Axl receptor tyrosine kinase in cell death control.. EMBO J.

[40] Burgel PR, Nadel JA (2008). Epidermal growth factor receptor-mediated innate immune responses and their roles in airway diseases.. Eur Respir J.

[41] Bernardo D, Garrote JA, Allegretti Y, León A, Gómez E (2008). Higher constitutive IL15R alpha expression and lower IL-15 response threshold in coeliac disease patients.. Clin Exp Immunol.

