# Supplementary figures and images for: Gliadin-Mediated Proliferation and Innate Immune Activation in Celiac Disease Are Due to Alterations in Vesicular Trafficking
Source: PLoS One. 2011 Feb 25;6(2):e17039. doi: 10.1371/journal.pone.0017039 (PMC3045409; doi:10.1371/journal.pone.0017039)

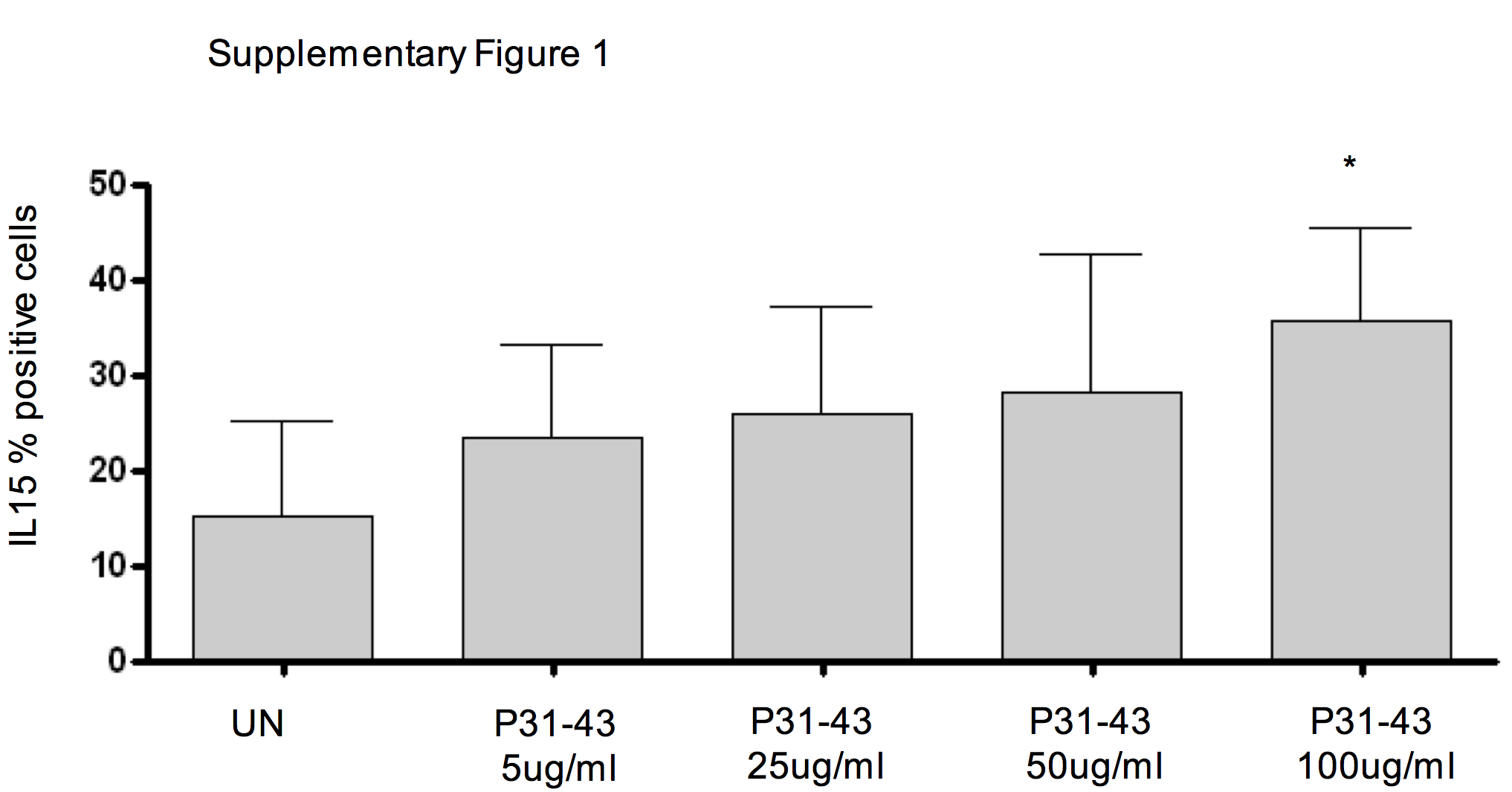

Supplement: Figure S1 — Dose-response effect of P31-43 treatment on IL-15 expression on CaCo-2 cell surfaces FACS analysis of IL-15 on Caco-2 cells surfaces after O/N treatment with varying concentrations of P31-43 peptide. UN = untreated. Columns indicate percentage of positive cells (mean and standard deviation of three independent experiments). *p<0.05 (Student's t-test). Optimised concentration of P31-43 for IL-15 expression on cell surface was 100 µg/ml. (TIF) [file pone.0017039.s001.tif]

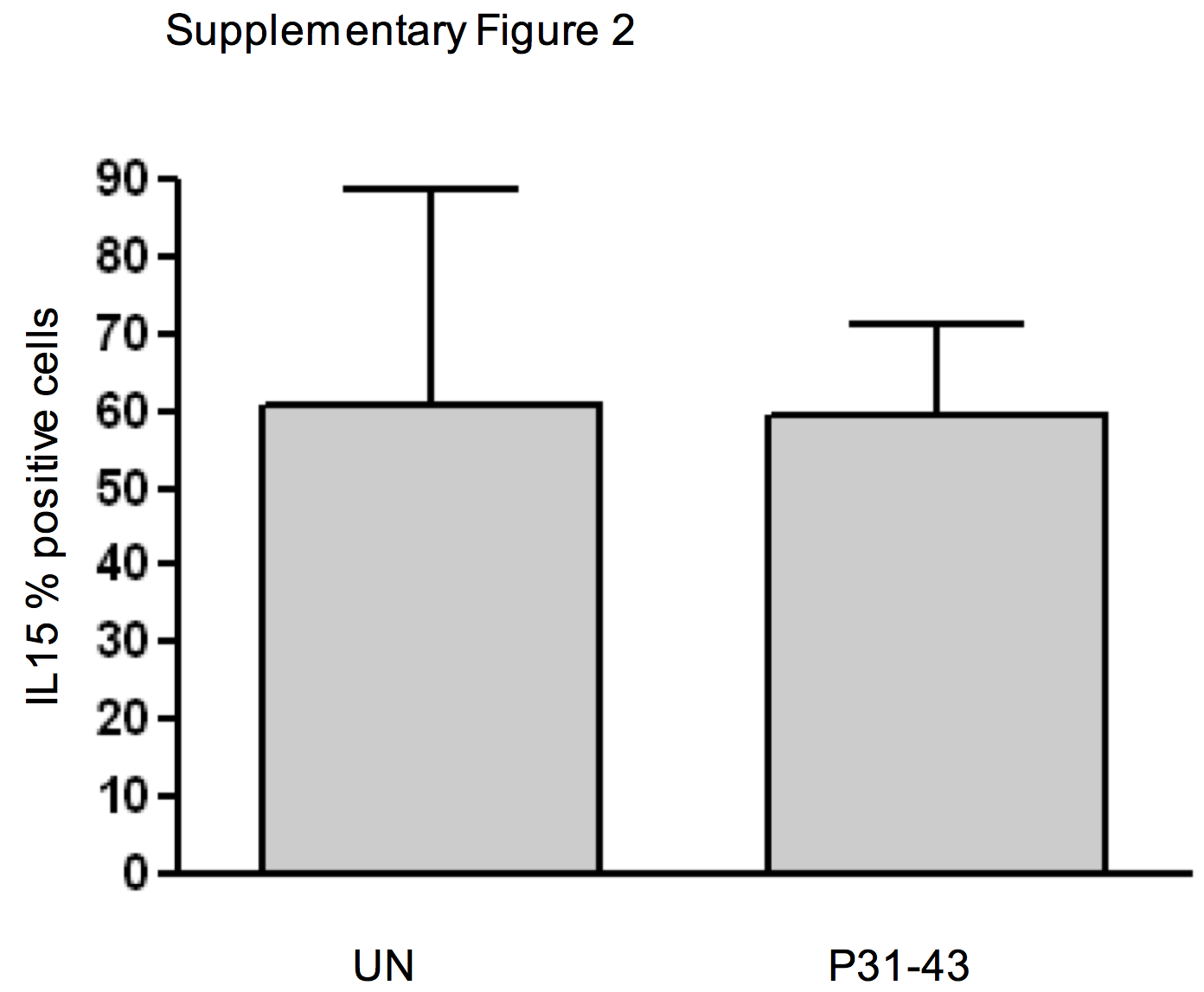

Supplement: Figure S2 — Overnight treatment with gliadin peptide P31-43 does not increase intracellular IL-15 expression. FACS analysis of IL-15 in the cytoplasm of CaCo-2 cells. Columns indicate percentage of positive cells (mean and standard deviation of four independent experiments). (TIF) [file pone.0017039.s002.tif]

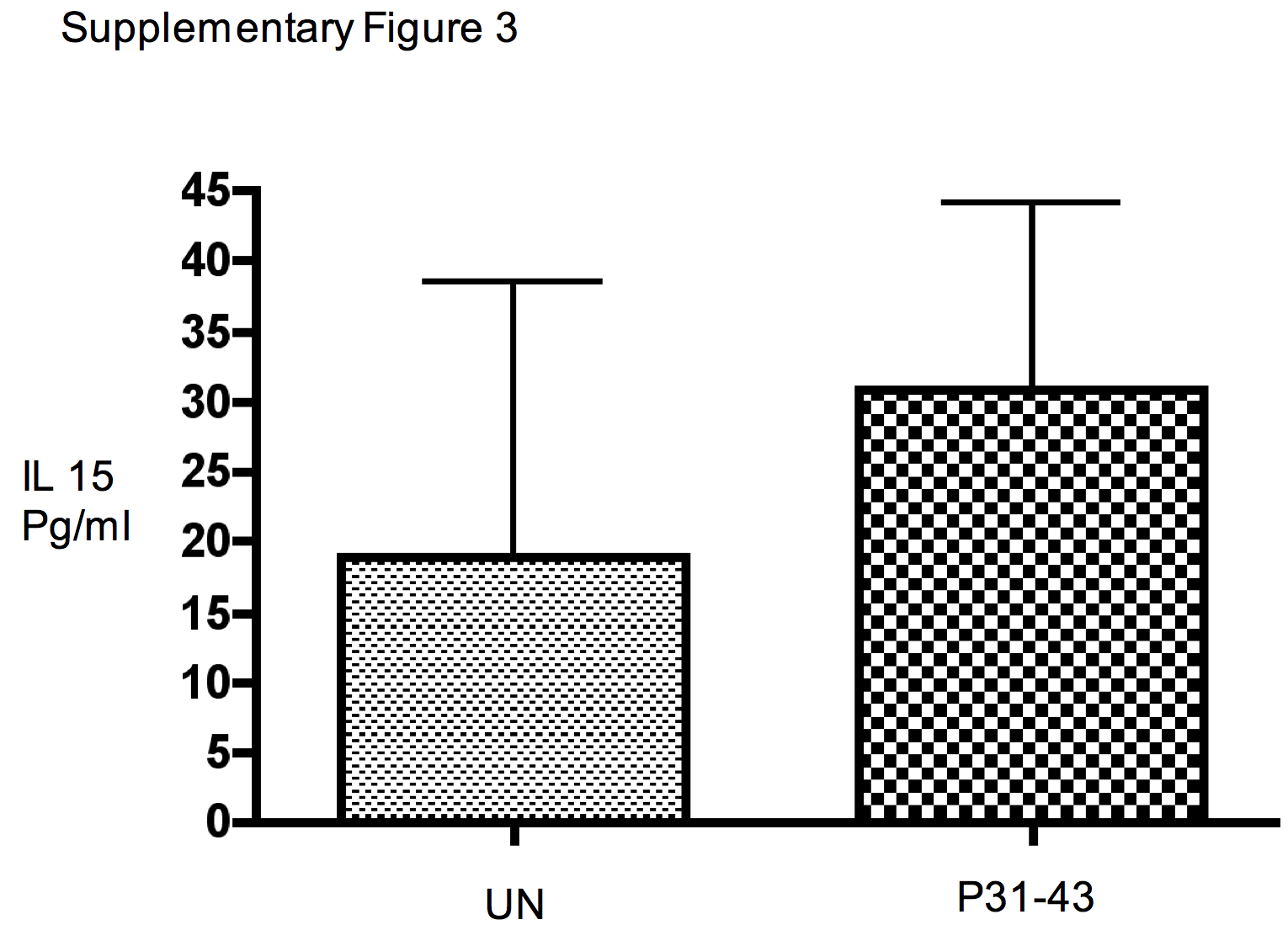

Supplement: Figure S3 — Overnight treatment with gliadin peptide P31-43 does not increase secreted IL-15. ELISA assay of IL-15 in medium of cultured CaCo-2 cells. Columns indicate pg/ml (mean and standard deviation of three independent experiments). (TIF) [file pone.0017039.s003.tif]

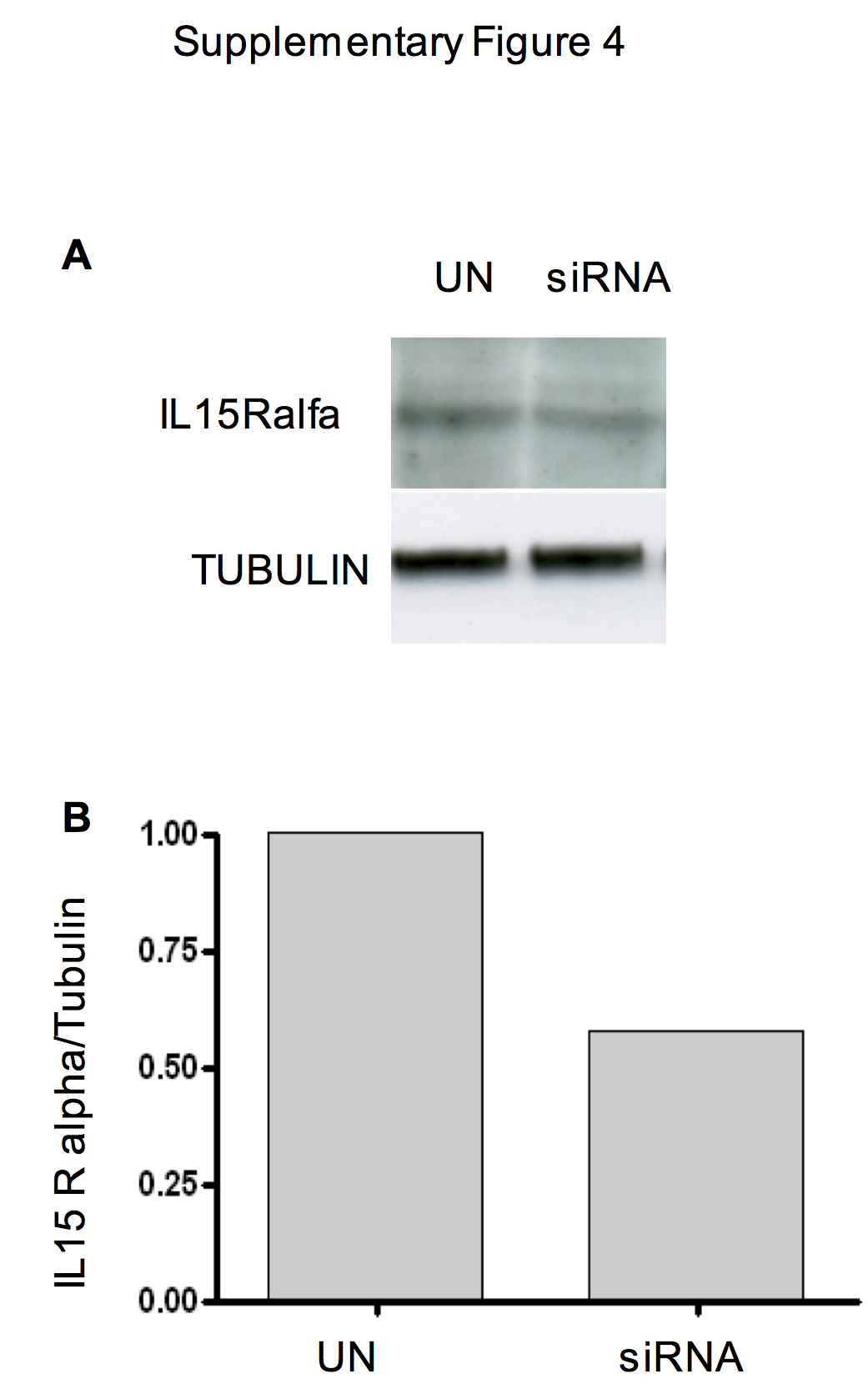

Supplement: Figure S4 — siRNA IL-15R alpha reduces IL-15R alpha protein expression. (A) CaCo-2 cells were transfected with IL-15R alpha siRNA, lysed and immunoblotted for IL-15R alpha expression. β-Tubulin was used as an internal control. (B) Densitometric analysis of IL-15R alpha expression compared to alpha-tubulin expression. The decrease (d) of IL-15R alpha was calculated as follows: dIL-15R = (IL-15R [t]/IL-15R [un])/(Tubulin [T]/Tubulin [UT]). Shown is one representative experiment out of three independent experiments. (TIF) [file pone.0017039.s004.tif]
