# Supplementary material for: Gliadin-Mediated Proliferation and Innate Immune Activation in Celiac Disease Are Due to Alterations in Vesicular Trafficking
Source: PLoS One. 2011 Feb 25;6(2):e17039. doi: 10.1371/journal.pone.0017039 (PMC3045409; doi:10.1371/journal.pone.0017039)
Supplement: Text S1 — Organ Culture Study. (RTF) [file pone.0017039.s005.rtf]

Text s1
Organ Culture Study
The intestinal samples were cultured for 24 h with medium alone or with P31-43 (100 μg/ml) or with peptic-tryptic gliadin peptides (PTG) (0.5 mg/ml) with or without blocking anti-IL-15 antibody (50 ng/ml) or blocking anti-EGFR antibody (2 mg/ml). All medium cultures were enriched with BrdU 10 μM (Sigma-Aldrich, Milan, Italy). Specimens were harvested, snap-frozen in liquid nitrogen, embedded in OCT and stored at –80°C until required. 
We used double immunofluorescence to evaluate crypt proliferation in 5 µm cryoStat sections from cultured biopsies. After a short (3 min) treatment with 1.5 N HCl, the sections were incubated with mouse monoclonal anti-BrdU 1:150 (GE Healthcare Amersham, Buckinghamshire, UK) for 1 h, followed by 30 min with secondary Alexa488-labelled anti-mouse IgG 1:150 (Invitrogen, San Giuliano Milanese, Italy) to identify BrdU-positive cells. After several washes in PBS, specimens were fixed with 3% paraformaldehyde (Sigma-Aldrich, Milan, Italy) for 5 min and incubated for 1 h with polyclonal rabbit anti-cow cytokeratin 1:50 (Dako, Glostrup Denmark) to stain epithelial cells. Slides were then covered for 30 min with Alexa633-labelled goat anti-rabbit immunoglobulins 1:200 (Invitrogen, San Giuliano Milanese, Italy), contrasted with Hoechst staining (Sigma-Aldrich, Milan, Italy) and then mounted in Mowiol4-88. All incubations were carried out at room temperature in a dark humid chamber. The number of BrdU-positive cells divided by the total number of cytokeratin-positive cells gave the percentage of BrdU-positive cells. 
