# Supplementary material for: Gliadin-Mediated Proliferation and Innate Immune Activation in Celiac Disease Are Due to Alterations in Vesicular Trafficking
Source: PLoS One. 2011 Feb 25;6(2):e17039. doi: 10.1371/journal.pone.0017039 (PMC3045409; doi:10.1371/journal.pone.0017039)
Supplement: Text S2 — RNA Extraction and Real-Time PCR. (RTF) [file pone.0017039.s006.rtf]

Text s2
RNA Extraction and Real-Time PCR 
cDNAs were generated from total RNA using the High Capacity cDNA Reverse Transcription Kit (Applied Biosystems, Foster City, CA). The resulting cDNA samples were subjected to a 10-cycle PCR amplification protocol followed by real-time PCR using TaqMan® PreAmp Master Mix Kit Protocol (Applied Biosystems, PN 4366127). Each TaqMan Gene Expression Assay consisted of two sequence-specific PCR primers and a TaqMan assay-FAM dye-labelled MGB probe. Eighty ng of total cDNA (as total input RNA) was used for each replicate assay. Three replicates were run for each sample in a 96-well plate format. The endogenous control gene used was beta-2-microglobulin (B2M). Assays were run with 2× Universal PCR Master Mix without UNG (uracil-N-glycosylase) on Applied Biosystems 7300 Real-Time PCR System using universal cycling conditions (10 min at 95°C; 15 sec at 95°C, 1 min 60°C, 40 cycles).
