# Supplementary material for: Gliadin-Mediated Proliferation and Innate Immune Activation in Celiac Disease Are Due to Alterations in Vesicular Trafficking
Source: PLoS One. 2011 Feb 25;6(2):e17039. doi: 10.1371/journal.pone.0017039 (PMC3045409; doi:10.1371/journal.pone.0017039)
Supplement: Text S3 — CTLL2 Proliferation Assays. (RTF) [file pone.0017039.s007.rtf]

Text s3
CTLL2 Proliferation Assays 
Cytotoxic T-cell line 2 (CTLL2) cells were analysed for proliferation in response to CaCo-2 cells treated with gliadin peptides. CTLL 2 cells were plated at a density of 0.3 x 10^5^ in 96-well round bottom plates together with 0.6 x 10^5^ (ratio 1:2) gamma-irradiated CaCo-2 cells, which had been previously pulsed overnight with medium alone, P31-43 (100 µg/ml), P31-43 and blocking anti-IL-15 (5 µg/ml) or P57-68 in 200 µl of complete medium. 
After 24 h of incubation, the cells were pulsed for 16 h with 1 µCi/well 3H-thymidine (Amersham-Pharmacia, Uppsala, Sweden). Radioactivity was assessed with a β counter (1600 TP, Hewlett Packard  California, San Francisco, USA).
