# Supplementary material for: Gliadin-Mediated Proliferation and Innate Immune Activation in Celiac Disease Are Due to Alterations in Vesicular Trafficking
Source: PLoS One. 2011 Feb 25;6(2):e17039. doi: 10.1371/journal.pone.0017039 (PMC3045409; doi:10.1371/journal.pone.0017039)
Supplement: Text S4 — Transferrin and Transferrin Receptor Analysis. (RTF) [file pone.0017039.s008.rtf]

Text s4
Transferrin and Transferrin Receptor Analysis
Texas Red-conjugated biferric-Transferrin (Molecular Probes) was used for pulse and chase experiments to highlight transferrin-positive vesicles and investigate colocalisation with IL-15-EGFP (42). CaCo 2 cells were  seeded on coverslips for 48 h and then transfected with IL-15-EGFP. After 48 h of transfection, they were pulsed for 15 min with Texas Red-conjugated biferric-transferrin (pulse phase). Coverslips were than washed and reincubated in growing media for 90 min (chase phase) in the presence of P31-43. The cells were than fixed and observed via microscopy after mounting (Zeiss LSM 510). 
For colocalisation analysis, the samples were examined with a Zeiss LSM 510 laser scanning confocal microscope. We used Argon/2 (458, 477, 488, 514 nm) and HeNe1 (543 nm) excitation lasers, which were switched on separately to reduce cross-talk of the two fluorochromes. The green and red emissions were separated by a dichroic splitter (FT 560) and filtered (515-to 540-nm band-pass filter for green and > 610-nm long pass filter for red emission). A threshold was applied to exclude approximately 99% of the signal found in control images. The weighted co-localisation coefficient represents the sum of intensities of the co-localising pixels in channels 1 and 2 as compared to the overall sum of pixel intensities above the threshold. This value could be 0 (no co-localisation) or 1 (all pixels co-localise). Bright pixels contributed more than faint pixels. The co-localisation coefficient represents the weighted co-localisation coefficients of Ch1 (red) with respect to Ch2 (green) for each experiment.[43]
Transferrin receptor expression on CaCo-2 cells was analysed by FACS analysis and immuno-fluorescence. 
For Facs analysis, CaCo-2 cells were plated in tissue culture dishes (35 x 10 mm) in 1.5 ml DMEM and 0.1% fetal calf serum and stimulated overnight at 37°C with 100 µg/μg/ml P31-43, 100 µgμg/ml P57-68 or medium alone. After 24 h, cells were scraped from the dishes at 4°C and transferred to a 96-well V-bottom plates (Costar Celbio, Milan, Italy). Flow cytometry analysis was performed as follows: after stimulation, 3-5x10^4^ cells were washed with PBS and labelled with unconjugated anti-Transferrin Receptor (Calbiochem, clone T56/14) mouse monoclonal antibody. Cells were incubated with the primary antibodies for 30 min at 4°C. After two washes with PBS, the cells were labelled with anti-mouse PE-conjugated secondary polyclonal antibody (Dako, Denmark, Polyclonal Rabbit anti-Mouse Immunoglobulins/PE) for 20 min at 4°C. After washing, the labelled cells were analysed on a FACSCalibur flow cytometer using CellQuestPro software (BD Bioscience, San Diego, California).
Transferrin receptor was stained on CaCo-2 cells with anti-Transferrin Receptor (Calbiochem, clone T56/14) mouse monoclonal antibody followed by secondary anti-mouse-Alexa-488 conjugated (Molecular probes, San Giuliano Milanese, Italy). The cells were seeded on coverslips and treated at 4°C, to block endocytosis, with anti-transferrin primary antibody for 45 min followed by staining for 45 min with secondary anti-mouse-Alexa-488. The cells were than fixed for 5 min with paraformaldehyde, mounted and observed.

Method for ELISA assay
IL-15 secretion was quantified in CaCo-2 culture supernatants after treatment with and without P31-43 by ELISA assay. Ninety-six-well plates (Immunoplate MaxiSorp, Nunc, Merelbeke, Belgium) were coated with anti-human IL-15 monoclonal antibody (BD Pharmingen) in 100 μl of carbonate buffer (0.1 M) overnight at 4°C. Wells were washed twice with PBS containing 0.05% Tween 20 after which samples were blocked by incubation with 200 μl of 2% BSA in PBS solution for two hours at room temperature. CaCo-2 supernatants were added (50 μl/well) and the wells were incubated for one hour at room temperature. After two washes, the wells were incubated with 50 μl of biotinylated anti-human IL-15 monoclonal antibody (BD Pharmingen) for 30 min at room temperature. Finally the wells were incubated with streptavidin-HRP (BD Pharmingen) diluted 1:5000 in PBS  containing 2% BSA. HRP was detected with the addition of 100 μl of TMB (3,3´,5,5´-tetramethylbenzidine) (Sigma-Aldrich, Milan, Italy). Absorbances were read on an ELISA reader at 450 nm.
